# Integrating rehabilitation services into primary health care: policy options for Iran

**DOI:** 10.1186/s12913-022-08695-8

**Published:** 2022-11-03

**Authors:** Saeed Shahabi, Carlotte Kiekens, Manal Etemadi, Parviz Mojgani, Ahmad Ahmadi Teymourlouei, Kamran Bagheri Lankarani

**Affiliations:** 1 Health Policy Research Center, Institute of Health, Shiraz University of Medical Sciences, Shiraz, Iran; 2 IRCCS MultiMedica, Milan, Italy; 3 National Center for Health Insurance Research, Tehran, Iran; 4 Iran-Helal Institute of Applied Science and Technology, Tehran, Iran; 5 Research Center for Emergency and Disaster Resilience, Red Crescent Society of The Islamic Republic of Iran, Tehran, Iran; 6 Department of Health Services Management, School of Health Management and Information Sciences, Iran University of Medical Sciences, Tehran, Iran

**Keywords:** Rehabilitation, Primary Health Care, Health Policy, Iran

## Abstract

**Background:**

Providing rehabilitation services in primary health care (PHC) is associated with numerous health, social, and economic benefits. Therefore, low and middle-income countries, such as Iran, should benefit from the advantages of integrating rehabilitation services into PHC. We conducted a qualitative study to determine policy solutions that could facilitate the integration of rehabilitation services into Iran’s PHC network.

**Methods:**

Semi-structured interviews were conducted with 38 participants, including health policymakers, rehabilitation managers, faculty members, and rehabilitation practitioners. Purposive and snowball sampling strategies were adopted to recruit participants. The WHO Health System building blocks framework analysis was applied to analyze the collected data.

**Results:**

Participants’ perspectives and experiences outlined potential policy options including: (1) stewardship: increasing political support, strengthening the leadership of the rehabilitation sector, and promoting inter-sectoral collaborations; (2) service delivery: increasing the knowledge of healthcare professionals, using local volunteers, deploying mobile rehabilitation teams, using telerehabilitation, and improving referral pathways; (3) financing: increasing government funding, preparing a package of rehabilitation services, and using appropriate payment mechanisms; (4) human resources: expanding rehabilitation workforce, training rehabilitation assistants, and enhancing employment and social opportunities; (5) information systems: establishing a comprehensive information system and an effective surveillance system; and (6) technologies: facilitating access to a range of rehabilitation equipment and raw materials, especially for prosthetics and orthotics services.

**Conclusion:**

Based on the WHO six building blocks framework, this study identified several policy options for integrating rehabilitation services into the Iranian PHC Network. Some of the policy options include increasing political support, promoting inter-sectoral collaborations, increasing the skills and knowledge of healthcare workers, establishing effective referral pathways, strengthening team-working, and increasing government funding.

## Background

In recent decades, demographic changes and improvements in acute healthcare have led to an increase in long-term conditions [1, 2]. The world’s population is aging as the number of people over the age of 60 years is projected to double by 2050 [3]. Moreover, the prevalence of non-communicable neurological conditions such as stroke, spinal cord injury, multiple sclerosis, and Parkinson’s disease is increasing [4]. Functional limitations and disability accompany these conditions. In addition, injuries caused by events such as traffic collisions, natural disasters, and industrial incidents have led to an increase in long-term musculoskeletal conditions, which affect nearly 1.3 billion people worldwide [5]. As a result, there is a significant demand for properly resourced rehabilitation services.

Rehabilitation services seek to empower people living with disability to participate in their communities by improving functional ability and reducing environmental barriers [6, 7]. Rehabilitation services play a significant role in reducing hospitalization, reducing health system costs, and augmenting economic productivity at the community level [8–10]. The World Health Organization (WHO) has always emphasized the need to provide rehabilitation services to the entire population, and introduced it as an essential part of universal health coverage (UHC) [11]. This approach requires strengthening rehabilitation services within all health system levels, including in primary health care (PHC) [7].

PHC services include diagnosing health conditions, recognizing functional problems, and referring patients for secondary and tertiary levels of care [7]. Modern PHC services go beyond the traditional care provided by a primary care physician, with an emphasis on disease prevention, detecting health conditions at an early-stage, and promoting health through community-based health and social programs [12]. PHC requires a multi-professional, interdisciplinary approach that includes physicians, nurses, dieticians, and rehabilitation professionals [7]. Providing rehabilitation services in PHC, besides health benefits, has social and economic benefits [13]. Due to the preventive effects of several rehabilitation interventions, their timely provision to targeted populations (such as disabled children or older people) can prevent secondary complications [14, 15]. In addition, early rehabilitation intervention can minimize the disability associated with long-term conditions such as stroke, cerebral palsy, and low back pain [7, 16] and optimize the consequences of other therapeutic interventions, such as surgery, by facilitating recovery [13]. Therefore, integrating rehabilitation services into PHC is essential, especially in low and middle-income countries such as Iran.

The concept of integration of health services as opposed to fragmented care is not new [17], and integrated care has been defined by several authors [18]. However, a principal characteristic of the concept of integrated care is bringing together key aspects in the design and delivery of care systems that are disjoined [19] to develop better health systems [17]. Integrated health services deliver a continuum of care, including disease prevention, treatment, and rehabilitation. Integration of services is important for achieving UHC [20, 21]. It has been shown that despite the extensive literature, the development of integrated PHC may not be a priority on a country’s health policy agenda and its establishment requires significant changes in a nation’s health system [22]. The Global Conference on Primary Health Care, in Astana, Kazakhstan in 2018, explored how primary health care could effectively and beneficially integrate the delivery of health services [23].

Iran’s Ministry of Health and Medical Education (MOHME) is the main provider and policymaker of health services. More than 90% of Iranians are covered by social insurance organizations for their healthcare, including the Iran Health Insurance Organization, the Social Security Organization, and the Armed Forces Health Insurance Organization [24]. The MOHME mainly finances and delivers primary healthcare, whereas public secondary and tertiary care services are financed through insurance schemes. The private sector plays a significant role in healthcare provision in Iran and mainly focuses on secondary and tertiary healthcare services in urban areas [25]. The primary healthcare system in rural areas functions well, but only meets the healthcare needs of a minority of the country’s population as most people in Iran live in the main cities. Fragmentation of the administration of services and categorizing them into primary healthcare, outpatient services, and inpatient services have resulted in the failure to provide an integrated and comprehensive system of health services [26].

**Fig. 1:**
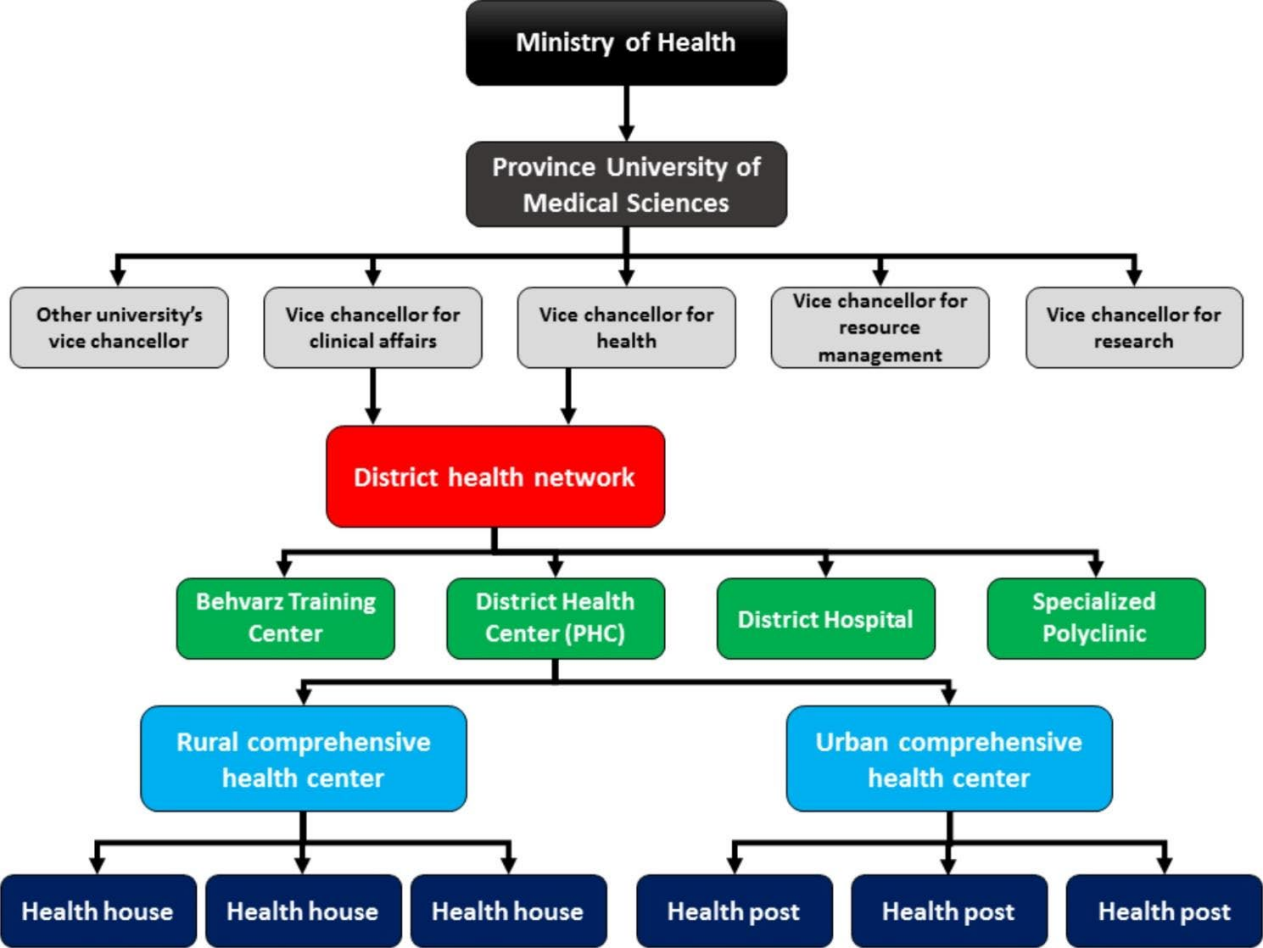
The structure of Iranian primary health care

The first steps to establishing a PHC network in Iran were taken in 1979, and the program was finally fully established in 1985 [27]. Evidence suggests that Iran’s PHC network is one of the most successful systems globally, and it has significantly promoted health indicators across the country, especially in rural areas [28, 29]. In the PHC network in Iran, the smallest health service unit is called the “health-house”, which is a facility staffed with trained healthcare providers called *Behvarz* [27]. A health-house covers the inhabitants of one village (and sometimes adjacent villages) up to a population of about 1,200 people. In densely populated villages, in addition to health-houses, there are rural health centers with a trained physician and a team of health workers, covering a population of about 7,000 [27]. In urban areas, health posts in addition to health centers provide similar services. The district health centers direct this network as the smallest administrative unit of the Iranian health system. The management of the networks at provincial and national levels is the responsibility of the universities of medical sciences and the MOHME, respectively [30]. PHC in Iran is mainly funded and provided by the government. All PHC services in Iran are free of charge and accessible to everyone so PHC coverage in rural areas is reported to be more than 95% [31]. However, the lack of clear criteria in payment systems, inadequate capitation fees and their inappropriate allocation, and the dominance of salary and fee-for-service payments are weaknesses of the payment system in the Iranian PHC network [27]. Figure 1 demonstrates the structure of the PHC network in Iran.

Despite the significant successes of Iran’s PHC network and the implementation of numerous reforms in recent decades [29], the program still faces challenges, including the lack of effective integration of rehabilitation services [32–34]. According to the recent recommendations of WHO on strengthening rehabilitation services in health systems and the increased demand for rehabilitation services in Iran due to aging population, high prevalence of non-communicable diseases, musculoskeletal conditions, traffic collisions, and natural disasters in recent years [7, 11, 35], we conducted this qualitative study to determine policy solutions that might facilitate the integration of rehabilitation services into Iran’s PHC Network. This study aimed to explore the current challenges and potential policy solutions for integrating rehabilitation services into the PHC system in Iran.

## Methods

This qualitative study was carried out in 2019 in Iran. The proposal for this study was reviewed and approved by the institutional review board of the Iran University of Medical Sciences, Tehran, Iran [36]. The standards for reporting qualitative research (SRQR) [37] and the consolidated criteria for reporting qualitative research (COREQ) [38] were followed in the development and reporting of this study.

### Participants sampling and recruiting

Health policymakers, rehabilitation managers, and rehabilitation faculty members with at least three years of experience, as well as allied healthcare professionals (physiotherapists, orthotists/prosthetists, and occupational therapists) with at least four years of experience, were considered as potential participants. Both purposive and snowball sampling strategies were adopted to select participants to ensure maximum variety in terms of job status, experience, specialty, and gender. Sampling and recruitment of participants continued until data saturation was reached, which was guaranteed by two sessions with duplicate findings. An invitation letter containing general information and study objectives was sent to participants. With the participant’s agreement, an appropriate time and place for the interview were set. Interviews were conducted face-to-face, by telephone, and virtually, depending on the availability of the participant.

### Data collection

Semi-structured interviews were conducted by the first author (S.SH) using a previously piloted interview guide. The research team revised the questions based on the feedback from the first interviews to improve transparency and clarity. The interview guide included three questions regarding integrating rehabilitation services into PHC: (1) What is the current status of rehabilitation services in PHC? (2) What are the most important barriers to integrating rehabilitation services into PHC? and (3) What are your suggested solutions to promote the integration of rehabilitation services into PHC? The sessions were held in a quiet environment without a third person. The interviews were audio recorded, and immediately after each session, the interviewer transcribed the recorded file. Files related to each interviewee were saved under a pseudonym to guarantee anonymity. Data were collected from January 2019 to June 2019.

### Data analysis

Framework analysis was applied to analyze the collected data [39]. Two authors (S.SH and A.A.T) performed the data analysis. Based on Braun and Clarke’s approach [40], the same authors read the transcribed texts frequently and independently following initial familiarization with the raw data. Then, preliminary codes were assigned to explain the meaning units. In the next step, the detected codes were abstracted by the research team and allocated to pre-defined themes according to the WHO health system six building blocks framework (stewardship, service delivery, financing, human resources, information systems, and medicines and technologies) [41]. Disagreements between authors were resolved through discussion and, in some cases, by using expert researcher advice. The data analysis was conducted manually and simultaneously with the data collection. Several authors with different scientific and professional backgrounds were involved in this step to minimize the potential risk of bias.

### Trustworthiness

Several strategies were adopted to cover credibility, dependability, transferability, confirmability, and authenticity [42]. Cross-checking by co-authors and data triangulation were used to enhance credibility. The involvement of several authors in data analysis increased the dependability of the result. Transferability was promoted by in-depth descriptions of participants and explaining the sampling process. To meet confirmability, member-checking by co-authors and participants was adopted to ensure that interpretations and findings were correctly extracted from the data. Finally, to ensure authenticity, excerpts from most participants were inserted to support the emerging themes.

### Ethical considerations

The ethical committee of the Iran University of Medical Sciences approved the study. Participants were aware that their participation was voluntary and that they could leave the study freely at any stage. An informed consent form was signed by participants before each interview.

## Results

The demographics of the 38 individuals who were interviewed are presented in Table 1. Two individuals (a female practitioner and a male faculty member) declined to participate in the study. From the data analysis, several policy solutions were suggested based on the WHO six building blocks framework. In Table 2, these proposed policy solutions have been explained in detail, along with participants’ direct quotes.

**Table 1:** The characteristics of participants

ID	sex	Age (years)	Specialty	Job	Interview format
**HP01**	Male	49	Health policy	Senior advisor of MoHME	Face-to-face
**HP02**	Male	49	Health policy	Rehabilitation deputy of WO	Telephone
**HP03**	Female	33	Health insurance	Deputy of IHIO	Telephone
**HP04**	Male	51	Health policy	Advisor of MoHME	Face-to-face
**HP05**	Male	49	Physician	SSO	Face-to-face
**HP06**	Male	-	Physician	MoHME	Face-to-face
**HP07**	Male	39	Health insurance	IHIO	Face-to-face
**HP08**	Female	29	Health insurance	Private insurer	Face-to-face
**HP09**	Male	44	Health technology assessment	Medical university	Face-to-face
**HP10**	Female	39	Health policy	SSO	Telephone
**HP11**	Female	36	Health policy	IHIO	Face-to-face
**RM01**	Male	53	Physiotherapy	Rehabilitation deputy of RC	Telephone
**RM02**	Male	49	Physician	Rehabilitation deputy of WO	Telephone
**RM03**	Female	29	Physiotherapy	SI	Telephone
**RM04**	Male	48	Physiotherapy	Rehabilitation deputy of WO	Telephone
**RM05**	Male	46	Occupational therapy	Rehabilitation deputy of WO	Telephone
**RM06**	Male	43	Physiotherapy	-	Telephone
**RM07**	Female	35	Prosthetics/Orthotics	Rehabilitation administrator	Face-to-face
**FM01**	Male	45	Prosthetics/Orthotics	Faculty member	Face-to-face
**FM02**	Male	37	Physiotherapy	Faculty member	Telephone
**FM03**	Male	36	Occupational therapy	Faculty member	Telephone
**FM04**	Male	57	Health policy	Faculty member	Face-to-face
**FM05**	Male	62	Physiotherapy	Faculty member	Telephone
**FM06**	Female	52	Occupational therapy	Faculty member	Telephone
**FM07**	Female	42	Prosthetics/Orthotics	Faculty member	Face-to-face
**FM08**	Male	58	Physiotherapy	Faculty member	Face-to-face
**P01**	Female	28	Physiotherapy	Physiotherapist	Telephone
**P02**	Female	27	Physiotherapy	Physiotherapist	Telephone
**P03**	Female	35	Physiotherapy	Physiotherapist	Telephone
**P04**	Female	29	Physiotherapy	Physiotherapist	Face-to-face
**P05**	Male	26	Physiotherapy	Physiotherapist	Face-to-face
**PO01**	Male	29	Prosthetics/Orthotics	Orthotist	Face-to-face
**PO02**	Female	29	Prosthetics/Orthotics	Prosthetist/Orthotist	Telephone
**PO03**	Male	30	Prosthetics/Orthotics	Orthotist	Telephone
**PO04**	Female	31	Prosthetics/Orthotics	Orthotist	Face-to-face
**OT01**	female	28	Occupational therapy	Hand therapist	Face-to-face
**OT02**	Male	29	Occupational therapy	Occupational therapist	Face-to-face
**OT03**	Female	36	Occupational therapy	Occupational therapist	Telephone

MOHME, Ministry of Health and Medical Education; WO, Welfare Organization; IHIO, Iranian Health Insurance Organization; SSO, Social Security Organization; RC, Red Crescent; SI, Supplementary Insurer.

**Table 2:** Proposed policy solutions regarding each block of WHO six building blocks framework

WHO six building blocks framework	Policy solutions	Quotes
**Stewardship**	Increasing political support	*“In order for these services to be considered at all levels of the health system, the leadership in this area needs to be strengthened, which requires political support. It is this political support that can provide the necessary basis for the growth of the field of rehabilitation.“[Health Policymaker 02]*
	Strengthening the leadership	
	Promoting inter-sectoral collaborations	*“Various organizations are involved in financing and providing rehabilitation services. The welfare organization itself provides community-based rehabilitation services. However, if there is close cooperation between this institution and the Ministry of Health, these services can be integrated into primary health care.“[Faculty member 01]*
	Increasing the awareness of policymakers	*“Interestingly, many health experts are still unaware that these [rehabilitation] services can be preventative and prevent many serious complications in the future. They always think that if treatment and therapy interventions fail, they should go to rehabilitation!“[Physiotherapist 03]*
	Participation of rehabilitation professionals in policy-making	*“What do you expect from a policymaker who has very little information about rehabilitation services? In my opinion, rehabilitation specialists should be involved in all level of health systems in order to improve their rehabilitation position.“[Faculty member 04]*
	Adhering to existing laws	*“Adherence to existing upstream laws and documents can be very effective in integrating rehabilitation services. Unfortunately, there is no good adherence to the existing laws.“[Health policymaker 03]*
	Strengthening NGOs and local communities	*“Recipients of rehabilitation services are usually disadvantaged and vulnerable groups and therefore do not have enough power to put pressure on policymakers. I think that by holding rehabilitation campaigns and associations, their needs can be better shown.“[Orthotist/Prosthetist 02]*
	Promoting the position of scientific rehabilitation societies	*“Scientific associations can provide a list of rehabilitation services that have preventive effects to consider in primary health care.“[Faculty member 05]*
**Services delivery**	Increasing the skills and knowledge of health care workers	*“We can teach some skills to current staff [Behvarzs] and ask them to provide basic musculoskeletal assessments as well as some training and exercises.“[Faculty member 02]*
	Using local volunteers	*“Many volunteers are interested in cooperating, especially in deprived and rural areas. With short training courses, they can be prepared to perform a set of tasks.“[Rehabilitation expert 01]*
	Using mobile rehabilitation teams	*“Rehabilitation professions can be used on a mobile basis. For example, visit the covered areas on a weekly basis and provide the necessary services.“[Health policymaker 05]*
	Applying telerehabilitation	*“We now have good internet throughout the country that we can use to provide telerehabilitation services.“[Rehabilitation expert 04]*
	Effective referral pathways	*“Existence of an effective referral system is a prerequisite for proper integration of rehabilitation services into health care, especially PHC.“[Health policymaker 10]*
	Team-working	*“There are many professions involved in PHC, and if there is no team-working, the desired goals will not be achieved at all!“[Health policymaker 07]*
	Clarifying the role of different professions	*“Proper integration of these services into PHC requires a clear understanding of the role of rehabilitation professions alongside other professions. Also, the knowledge of physicians and other health care workers should be enhanced in relation to rehabilitation services in order to form a good team-working“[Faculty member 06]*
**Financing**	Increasing the government funding	*“If we want to consider rehabilitation services in primary care, we need new financial resources. The government should allocate more resources to this area.“[Health policymaker 03]*
	Preparing a package of rehabilitation services	*“Some physiotherapy services can be provided to pregnant mothers during pregnancy. There are also rehabilitation services for infants and children that, if provided in a timely manner, can prevent many problems in the future.“[Physiotherapist 03]*
	Considering competitive payment mechanisms	*“How you pay employees is very important, both to the rehabilitation professionals and to current PHC staff. [Payment] Mechanisms should be used to motivate them to perform their tasks.“[Health policymaker 05]*
**Human resources**	Increasing the training of the rehabilitation workforce	*“If we want to provide these services well in primary health care, we need to train more rehabilitation professionals in the fields of physiotherapy, occupational therapy and prosthetics/orthotics.“[Faculty member 02]*
	Training rehabilitation assistants	*“Many rehabilitation graduates are not interested in working in PHC, especially in deprived and remote areas. Rehabilitation assistants can be trained by taking two-year courses.“[Faculty member 04]*
	Improving the job and social opportunities	*“Graduates will no longer be interested in working in the PHC network until we improve the occupational and social status of the workforce in disadvantaged and remote areas.“[Physiotherapist 02]*
**Health information**	Creating a comprehensive information system	*“How can we have an effective intervention if we do not have an information system of the needs and records of the patient?“[Faculty member 04]*
	Establishing an effective surveillance system	*“There should be a comprehensive system that can well address the rehabilitation needs of the covered population.“[Health policymaker 07]*
**Medicines and technology**	Providing needed equipment and raw materials	*“Some of the required equipment can be provided to employees in remote and rural areas, such as goniometers to measure skeletal deviations and range of motion… Also, some equipment is really expensive and it is better to be in the city or province“[Physiotherapist 03]*

### Stewardship

During the interviews, the need to increase political support and strengthen the leadership in the field of rehabilitation in Iran was repeatedly mentioned. One of the participants said:

*“For these services to be considered at all levels of the health system, the leadership in this area needs to be strengthened, which requires political support. It is this political support that can provide the necessary basis for the growth of the field of rehabilitation.“[Health Policymaker 02]*

Participants emphasized the promotion of intersectoral collaborations as one of the potential solutions. Participants believed that due to the interdisciplinary nature of rehabilitation, several stakeholders are involved in providing these services, requiring inter-sectoral co-operation.

*“Various organizations are involved in financing and providing rehabilitation services. The welfare organization itself provides community-based rehabilitation services. However, if there is close co-operation between this institution and the Ministry of Health, these services can be integrated into primary healthcare.“[Faculty member 01]*

The level of awareness of policy- and decision-makers about the effects of rehabilitation services, especially their preventive effects, should be increased. Many relevant policymakers view these services as tertiary interventions and a strategy when other options have failed.

*“Interestingly, many health experts are still unaware that these [rehabilitation] services can be preventative and prevent many serious complications in the future. They always think that they should go to rehabilitation if treatment and therapy interventions fail!“[Physiotherapist 03]*

Participation of rehabilitation professionals in the policy-making process can facilitate the strengthening of rehabilitation services, especially in PHC. One participant said:

*“What do you expect from a policymaker with very little information about rehabilitation services? In my opinion, rehabilitation specialists should be involved in the care processes of care services to improve their rehabilitation position.“[Faculty member 04]*

Participants believed that national policy documents emphasized the provision of rehabilitation services in healthcare. They suggested that adhering to, and strengthening existing statutes could provide the necessary basis for strengthening rehabilitation services in PHC.

*“Adherence to existing upstream laws and documents can be very effective in integrating rehabilitation services. Unfortunately, there is no good adherence to the existing laws.“[Health policymaker 03]*

Individual service recipients, local community organizations and non-governmental organizations can campaign to improve services:

*“Recipients of rehabilitation services are usually disadvantaged and vulnerable groups and therefore do not have enough power to pressure policymakers. I think their needs can be better shown by holding rehabilitation campaigns and associations.“[Orthotist/Prosthetist 02]*

Rehabilitation associations can also play a crucial role in demonstrating the effectiveness of rehabilitation interventions, and informing relevant policy makers:

*“Scientific associations can provide a list of rehabilitation services that have preventive effects to consider in primary health care.“[Faculty member 05]*

### Service delivery

Some participants proposed increasing the skills and knowledge of current healthcare workers to provide a range of rehabilitation services in PHC. A university professor believed that:

*“We can teach some skills to current staff [Behvarzs] and ask them to provide basic musculoskeletal assessments and some training and exercises.“[Faculty member 02]*

Given the current workload of health workers, suitably trained local volunteers could provide a range of rehabilitation services.

*“Many volunteers are interested in cooperating, especially in deprived and rural areas. They can be prepared to perform a set of tasks with short training courses.“[Rehabilitation expert 01]*

Using mobile rehabilitation teams was mentioned as another solution. Participants believed that mobile rehabilitation teams could provide rehabilitation services, especially in rural and remote areas due to the lack of specialized personnel in non-urban settings.

*“Rehabilitation professionals can serve in mobile clinics. For example, one may visit the covered areas every week and provide the necessary services.“[Health policymaker 05]*

Telerehabilitation could be used to facilitate the provision of rehabilitation services to remote areas. The development of the Internet across the country could greatly facilitate this approach.

*“We now have good Internet throughout the country that we can use to provide telerehabilitation services.“[Rehabilitation expert 04]*

Many participants described how rehabilitation services could be well integrated into PHC if there were effective referral pathways.

*“Existence of an effective referral system is a prerequisite for proper integration of rehabilitation services in healthcare, especially primary health care.“[Health policymaker 10]*

As different professions are involved, team working was emphasized as one of the most important strategies for the effective integration of rehabilitation services.

*“There are many professions involved in primary health care, and if there is no team-working, the desired goals will not be achieved!“[Health policymaker 07]*

Nonetheless, clarity on the role of professions involved in PHC, including allied health professionals, and adequate knowledge of physicians and other healthcare workers about rehabilitation professions, can facilitate team working. In this regard, one of the participants said:

*“Proper integration of these services in primary health care requires a clear understanding of the role of rehabilitation professions alongside other professions. Also, the knowledge of physicians and other healthcare workers should be enhanced about rehabilitation services to form a good team-working“[Faculty member 06]*

### Financing

Integration of rehabilitation services into PHC requires an increase in government funding as a health policymaker said:

*“If we want to consider rehabilitation services in primary care, we need new financial resources. The government should allocate more resources to this area.“[Health policymaker 03]*

An interesting point raised was the need to provide a package of rehabilitation interventions that can be integrated into PHC, such as preventive interventions for pregnant mothers, infants, and children.

*“Some physiotherapy services can be provided to pregnant mothers during pregnancy. There are also rehabilitation services for infants and children that, if provided timely, can prevent many future problems.“[Physiotherapist 03]*

Many participants believed that having appropriate and competitive payment mechanisms for the PHC workforce could lead to more effective rehabilitation services provided by PHC staff.

*“How you pay employees is very important to rehabilitation professionals and current primary health care staff. [Payment] Mechanisms should be used to motivate them to perform their tasks.“[Health policymaker 05]*

### Human resources

The workforce is one of the most critical challenges addressed in this study. Several solutions were proposed, including increasing the training of the rehabilitation workforce.

*“If we want to provide these services well in primary health care, we need to train more rehabilitation professionals in physiotherapy, occupational therapy, and prosthetics/orthotics.“[Faculty member 02]*

A curriculum could be developed to train rehabilitation assistants who would be able to work in PHC.

*“Many rehabilitation graduates are not interested in working in primary health care, especially in deprived and remote areas. Rehabilitation assistants can be trained by taking two-year courses.“[Faculty member 04]*

Nonetheless, even if the rehabilitation workforce were increased, there is still a lack of interest amongst rehabilitation professionals in engaging in PHC due to inadequate employment and social opportunities in disadvantaged areas. Therefore, job and social opportunities should be improved for deprived and less privileged areas.

*“Graduates will no longer be interested in working in the primary healthcare network until we improve the occupational and social status of the workforce in disadvantaged and remote areas.“[Physiotherapist 02]*

### Health information

The need for a comprehensive information system was mentioned to facilitate patient follow-up and improve referral pathways in PHC.

*“How can we have an effective intervention if we do not have an information system of the needs and records of the patient?“[Faculty member 04]*

An effective surveillance system that can prepare accurate and timely information on the rehabilitation needs of the population could provide the evidence base for rehabilitation services in PHC.

*“There should be a comprehensive system that can well address the rehabilitation needs of the covered population.“[Health policymaker 07]*

### Medicines and technologies

Providing effective rehabilitation services requires equipment and materials. In this regard, some participants believed that basic equipment and materials could be provided at the rural level and more expensive and sophisticated equipment could be provided at city or provincial levels.

*“Some of the required equipment can be provided to employees in remote and rural areas, such as goniometers to measure skeletal deviations and range of motion... Also, some equipment is really expensive and it is better to be in the city or province“[Physiotherapist 03]*

## Discussion

Rehabilitation services have been a key but forgotten component of PHC in planning and providing health services. Although these services play an important role in all levels of care, their focus should be close to where people live and work to facilitate their utilization [43]. At present, rehabilitation services are provided at higher levels of care, and this type of fragmented rehabilitation service is not efficient or cost-effective. Nonetheless, integration of these services into PHC, as a low-cost patient-centered level of care, will lead to the recovery of more patients with higher cost-effectiveness [44].

The first key finding of this study was to design an over-arching concept for integrating rehabilitation services into PHC with respect to legal frameworks, inter-sectoral co-operation, and participation of NGOs and scientific societies. Indeed, effective integration of rehabilitation within the health system depends on the quality of rehabilitation stewardship and governance [11, 32]. Achieving the goal of rehabilitation as a key component of UHC and integrating it into the national health plan and budget, as outlined in the 1978 Alma-Ata and 2018 Astana Declarations, requires strong leadership and governance, adequate funding, efficient service delivery models, multidisciplinary rehabilitation workforce, affordable assistive equipment, and integration of rehabilitation data into the health information system [45].

Proposed policy options for providing rehabilitation services at the first level of care include telerehabilitation, mobile rehabilitation teams, and using local capacity to provide rehabilitation services. Previous studies have shown that in health systems where rehabilitation in the primary care infrastructure is very poor, telerehabilitation is a potentially effective way to compensate for this deficiency [46]. Collaboration and team-working were important pillars of the effective integration of rehabilitation services into PHC in the present study. Developing relationships between rehabilitation and primary care providers, enhancing interprofessional skills, including knowledge of professional roles, and building trust are among the critical issues needed for effective integration [47, 48]. Indeed, a proper understanding of the role of rehabilitation professionals in primary care by professionals in the field, other health personnel, and health system policymakers facilitates integration [49]. The WHO has described how a mobile multidisciplinary rehabilitation team at the primary care level with defined referral pathways and local assistants can be effective in providing rehabilitation services [7], especially in rural and remote areas, all three of which mentioned by participants in the present study. The experience of training and employing local health volunteers in Thailand to assist rehabilitation professionals as part of a health team improved the performance of people with disabilities in the community. It has been an effective policy, in line with our findings [50].

Based on the literature, inadequate financing is one of the most critical challenges of the rehabilitation sector in Iran [33]. The use of rehabilitation services and a disabled or elderly family member are known to be some of the main determinants when someone is facing catastrophic health expenditure [51, 52]. Unfortunately, in the reforms of recent decades in the Iranian health system, not enough attention has been paid to rehabilitation services. As a result, the private sector finances and provides a major part of these services [53, 54].

An important issue addressed in this study is financing the integration of rehabilitation services into PHC, which means increasing government investment in this area and increasing the utilization of such services by the public. The collaboration of donors and NGOs can be very effective in financing rehabilitation services in PHC in countries like Iran, which face limited financial resources [32, 33]. In relation to how payments are made to rehabilitation providers, applying the global budget approach to compensate for the services provided by PHC staff is more effective than approaches such as fee-for-service, in which the necessary resources to pay the rehabilitation service provider must be provided by the physician. With this approach, payment is made according to the volume of services provided, making service integration more complex [47]. Until now, there has not been any defined package of rehabilitation services to incorporate them into the basic health benefits package in Iran [8]. Evidence suggests that considering rehabilitation interventions with high-value care, such as services with preventive effects, can reduce the financial burden on the health system and help target groups participate effectively in the community [8, 55].

Effective integration of rehabilitation services into PHC depends on a trained rehabilitation workforce. However, in addition to the small number of rehabilitation graduates in many countries, especially developing countries [13], many graduates are not interested in serving in deprived and remote areas [7]. This situation is also seen in Iran (64.6 physiotherapists and 22.1 occupational therapists per 1,000,000 population), where the majority of graduates are working in metropolitan areas due to preferable social and economic conditions [33, 34]. One of the proposed solutions in the present study was to increase the number of graduates in the rehabilitation professions, although this policy needs time to be implemented. It is also necessary to encourage graduates to work in rural areas through providing attractive economic and social packages, which will take time to become effective. In the meantime, it may be possible to train rehabilitation assistants who can perform some of the basic tasks of the qualified rehabilitation professionals [7]. It may also be possible to provide basic training to local volunteers to assess rehabilitation needs and provide some essential rehabilitation services. This approach has been used by a community-based rehabilitation program in China [56], and local and community contributions have been effective in the integration of rehabilitation services into PHC into Chile [7].

The national health information system often does not include rehabilitation-related information. This hinders rehabilitation policy at all levels and is one of the challenges of strengthening rehabilitation services globally [57]. In Iran, the lack of data on physical access to infrastructure and rehabilitation resources in public and private centers is a significant policy challenge to increasing access to rehabilitation services [58]. Thus, a comprehensive information system that includes rehabilitation-related information is one of the prerequisites for integrating rehabilitation into healthcare services, especially PHC [57]. Furthermore, establishing an effective surveillance system can inform policy- and decision-makers about the target groups and the need for rehabilitation services in different parts of the country and provide the necessary basis for considering the required interventions in PHC [59].

The approach to integrating rehabilitation into UHC in each country reflects the characteristics of that country’s health system, its values, the horizontal development of the social welfare system, and the availability of resources and infrastructure [60]. Therefore, a single global policy for integrating rehabilitation in the health system, especially at the first level of healthcare, cannot be recommended and should be tailored to the context of each country. However, the policy options proposed in this study for integrating rehabilitation services into PHC in Iran can be considered by other low- and middle-income countries, as well as countries with a health system structure similar to Iran.

Despite all the efforts made, the present study has some limitations. Rehabilitation is a broad field consisting of many different professions, of which this study focused on only three: physiotherapy, occupational therapy, and prosthetics/orthotics. In addition, service users were not included in this study, missing important viewpoints.

## Conclusion

This study has outlined policy options for integrating rehabilitation services into the Iranian PHC network based on the WHO six building blocks framework. In the governance dimension, the leadership of integration, legislation, and participatory decision-making were shown to be the main components of integration policy. Legislation with sufficient executive guarantees is required to put this issue on the policy agenda of the Iranian health system. Considering all policy stakeholders, especially the private sector as the leading actor and provider of secondary and tertiary care rehabilitation services in Iran, the Welfare Organization as the primary steward of disability in the country, and especially the people’s representatives as the users of services and main stakeholders of this policy, can facilitate the integration of rehabilitation services into PHC.

## Data Availability

The datasets used and/or analysed during the current study are available from the corresponding author on reasonable request.

## Abbreviations

PHC: Primary health care
SRQR: The standards for reporting qualitative research
COREQ: the consolidated criteria for reporting qualitative research.

## References

[1] Bennett JE, Stevens GA, Mathers CD, Bonita R, Rehm J, Kruk ME (2018). NCD Countdown 2030: worldwide trends in non-communicable disease mortality and progress towards Sustainable Development Goal target 3.4. The Lancet.

[2] Khorrami Z, Rezapour M, Etemad K, Yarahmadi S, Khodakarim S, Hezaveh AM (2020). The patterns of non-communicable disease multimorbidity in Iran: a multilevel analysis. Sci Rep.

[3] Chen N, Fong DYT, Wong JYH (2022). Secular Trends in Musculoskeletal Rehabilitation Needs in 191 Countries and Territories From 1990 to 2019. JAMA Netw Open.

[4] Raggi A, Monasta L, Beghi E, Caso V, Castelpietra G, Mondello S, et al. Incidence, prevalence and disability associated with neurological disorders in Italy between 1990 and 2019: an analysis based on the Global Burden of Disease Study 2019. J Neurol. 2021:1–19. 10.1007/s00415-021-10774-5 PMC9938710 34498172

[5] Safiri S, Kolahi AA, Cross M, Hill C, Smith E, Carson-Chahhoud K (2021). Prevalence, Deaths, and Disability‐Adjusted Life Years Due to Musculoskeletal Disorders for 195 Countries and Territories 1990–2017. Arthritis Rheumatol.

[6] Gutenbrunner C, Nugraha B (2019). 2.1 Rehabilitation: rehabilitation as a health strategy. J Int Soc Phys Rehabil Med.

[7] World Health Organization (2018). Access to rehabilitation in primary health care: an ongoing challenge.

[8] Shahabi S, Pardhan S, Ahmadi Teymourlouy A, Skempes D, Shahali S, Mojgani P (2021). Prioritizing solutions to incorporate Prosthetics and Orthotics services into Iranian health benefits package: Using an analytic hierarchy process. PLoS ONE.

[9] World Health Organization (2017). WHO standards for prosthetics and orthotics.

[10] Shahabi S (2020). Economic evaluations of physical rehabilitation interventions in older adults with hip and/or knee osteoarthritis: a systematic review. Eur J Physiother.

[11] World Health Organization (2017). Rehabilitation in health systems.

[12] Espinosa-González AB, Normand C (2019). Challenges in the implementation of primary health care reforms: a qualitative analysis of stakeholders’ views in Turkey. BMJ open.

[13] World Health Organization. Rehabilitation in health systems: guide for action. World Health Organization; 2019.

[14] Kepenek-Varol B, Tanrıverdi M, İşcan A, Alemdaroğlu-Gürbüz İ (2019). The acute effects of physiotherapy on general movement patterns in preterm infants: a single-blind study. Early Hum Dev.

[15] Almousa S, Lamprianidou E, Kitsoulis G (2018). The effectiveness of stabilising exercises in pelvic girdle pain during pregnancy and after delivery: a systematic review. Physiother Res Int.

[16] Shahabi S, Shabaninejad H, Kamali M, Jalali M, Ahmadi Teymourlouy A (2020). The effects of ankle-foot orthoses on walking speed in patients with stroke: a systematic review and meta-analysis of randomized controlled trials. Clin Rehabil.

[17] Kodner DL, Spreeuwenberg C. Integrated care: meaning, logic, applications, and implications–a discussion paper. *Int J Integr Care* 2002, **2**. 10.5334/ijic.67 PMC1480401 16896389

[18] Armitage GD, Suter E, Oelke ND, Adair CE. Health systems integration: state of the evidence. Int J Integr Care. 2009, 9. 10.5334/ijic.316 PMC2707589 19590762

[19] Goodwin N. Understanding integrated care. Int J Integr Care. 2016, 16(4). 10.5334/ijic.2530 PMC5354214 28316546

[20] Cash-Gibson L. Integrating Health Services, Technical Series on Primary Health Care. 2018.

[21] Kluge H, Kelley E, Barkley S, Theodorakis PN, Yamamoto N, Tsoy A (2018). How primary health care can make universal health coverage a reality, ensure healthy lives, and promote wellbeing for all. The Lancet.

[22] Lionis C, Symvoulakis EK, Markaki A, Vardavas C, Papadakaki M, Daniilidou N, et al. Integrated primary health care in Greece, a missing issue in the current health policy agenda: a systematic review. Int J Integr Care. 2009, 9(3). 10.5334/ijic.322 PMC2748181 19777112

[23] World Health Organization. Declaration of Astana: Global Conference on Primary Health Care: Astana, Kazakhstan, 25 and 26 October 2018. Geneva: World Health Organization. 2019.

[24] Etemadi M, Gorji HA, Kangarani HM, Ashtarian K (2017). Power structure among the actors of financial support to the poor to access health services: Social network analysis approach. Soc Sci Med.

[25] Aryankhesal A, Etemadi M, Agharahimi Z, Rostami E, Mohseni M, Musavi Z (2016). Analysis of social functions in Iran’s public hospitals: pattern of offering discounts to poor patients. Int J Hum Rights Healthc.

[26] Danaei G, Farzadfar F, Kelishadi R, Rashidian A, Rouhani OM, Ahmadnia S (2019). Iran in transition. The Lancet.

[27] Tabrizi JS, Pourasghar F, Nikjoo RG (2017). Status of Iran’s primary health care system in terms of health systems control knobs: a review article. Iran J Public Health.

[28] Nekoei Moghadam M, Amiresmaili M, Sadeghi V, Zeinalzadeh AH, Tupchi M, Parva S (2018). A qualitative study on human resources for primary health care in Iran. Int J Health Plann Manage.

[29] Lankarani KB, Alavian SM, Peymani P (2013). Health in the Islamic Republic of Iran, challenges and progresses. Med J Islam Repub Iran.

[30] Shadpour K. Primary health care networks in the Islamic Republic of Iran. East Mediterr Health J, 6 (4), 822–825, 2000. 2000. 11794090

[31] Rahimi H, Haghdoost A, Noorihekmat S (2022). A qualitative study of challenges affecting the primary care system performance: Learning from Iran’s experience. Health Sci Rep.

[32] Shahabi S, Skempes D, Mojgani P, Bagheri Lankarani K, Heydari ST. Stewardship of physiotherapy services in Iran: common pitfalls and policy solutions. Physiother Theory Pract. 2021:1–14. 10.1080/09593985.2021.1898705 33760676

[33] Shahabi S, Mojgani P, Shabaninejad H, Teymourlouy AA, Behzadifar M, Lankarani KB (2021). Physical rehabilitation financing in Iran: a policy analysis using Kingdon’s multiple streams. BMC Health Serv Res.

[34] Abdi K, Arab M, Khankeh HR, Kamali M, Rashidian A, Farahani FK, et al. Challenges in providing rehabilitation services for people with disabilities in Iran: A qualitative study. J Adv Med Med Res. 2016:1–11. 10.19082/1476 PMC4700893 26767101

[35] Shahrezaee M, Keshtkari S, Moradi-Lakeh M, Abbasifard M, Alipour V, Amini S (2020). Burden of musculoskeletal disorders in Iran during 1990–2017: estimates from the Global Burden of Disease Study 2017. Arch Osteoporos.

[36] Shahabi S, Teymourlouy AA, Shabaninejad H, Kamali M, Lankarani KB, Mojgani P (2020). Physical rehabilitation in Iran after international sanctions: explored findings from a qualitative study. Global Health.

[37] O’Brien BC, Harris IB, Beckman TJ, Reed DA, Cook DA (2014). Standards for reporting qualitative research: a synthesis of recommendations. Acad Med.

[38] Tong A, Sainsbury P, Craig J (2007). Consolidated criteria for reporting qualitative research (COREQ): a 32-item checklist for interviews and focus groups. Int J Qual Health Care.

[39] Gale NK, Heath G, Cameron E, Rashid S, Redwood S (2013). Using the framework method for the analysis of qualitative data in multi-disciplinary health research. BMC Med Res Methodol.

[40] Braun V, Clarke V (2006). Using thematic analysis in psychology. Qual Res Psychol.

[41] World Health Organization (2010). Monitoring the building blocks of health systems: a handbook of indicators and their measurement strategies.

[42] Kyngäs H, Kääriäinen M, Elo S (2020). The trustworthiness of content analysis. The application of content analysis in nursing science research.

[43] Sherry K (2014). Disability and rehabilitation: Essential considerations for equitable, accessible and poverty-reducing health care in South Africa. S Afr Health Rev.

[44] Louw Q, Grimmer K, Dizon J, Machingaidze S, Parker H, Ernstzen D (2018). Building capacity in primary care rehabilitation clinical practice guidelines: a South African initiative. Health Res Policy Syst.

[45] Gimigliano F, Negrini S (2017). The World Health Organization” rehabilitation 2030: a call for action". Eur J Phys Rehabil Med.

[46] Skempes D, Kiekens C, Malmivaara A, Michail X, Bickenbach J, Stucki G. Supporting government policies to embed and expand rehabilitation in health systems in Europe: A framework for action. Health Policy. 2021. 10.1016/j.healthpol.2021.06.014 34281701

[47] McColl MA, Shortt S, Godwin M, Smith K, Rowe K, O’Brien P (2009). Models for integrating rehabilitation and primary care: a scoping study. Arch Phys Med Rehabil.

[48] Halle AD, Mroz TM, Fogelberg DJ, Leland NE (2018). Occupational therapy and primary care: Updates and trends. Am J Occup Ther.

[49] Maharaj S, Chung C, Dhugge I, Gayevski M, Muradyan A, McLeod KE (2018). Integrating physiotherapists into primary health care organizations: the Physiotherapists’ perspective. Physiother Can.

[50] Chinchai P, Khamwong P (2017). The effects of rehabilitation education for village health volunteers on walking speed and upper extremity function in stroke survivors in Thailand. S Afr J Occup Ther.

[51] Piroozi B, Moradi G, Nouri B, Bolbanabad AM, Safari H (2016). Catastrophic health expenditure after the implementation of health sector evolution plan: a case study in the west of Iran. Int J Health Policy Manag.

[52] Moradi G, Safari H, Piroozi B, Qanbari L, Farshadi S, Qasri H (2017). Catastrophic health expenditure among households with members with special diseases: A case study in Kurdistan. Med J Islam Repub Iran.

[53] Etemadi M, Ashtarian K, Ganji N, Kangarani HM, Gorji HA. Have the poor been considered in the Health Sector Evolution Plan? A qualitative study of the Iranian health system. Int J Hum Rights Healthc. 2019.

[54] Basakha M (2021). Economic Profile of Iranian Rehabilitation Services: 2002–2017. Archives of Rehabilitation.

[55] Yahyavi Dizaj J, Na’emani F, Fateh M, Soleimanifar M, Arab AM, Zali ME (2020). Inequality in the Utilization of Rehabilitation Services Among Urban and Rural Households in Iran: A Cross-Sectional Study. Archives of Rehabilitation.

[56] Chen S, Lei Y, Dai H, Wu J, Yang Z, Liao X (2020). Community-based rehabilitation service in Chengdu, Southwest China: a cross-sectional general survey. BMC Health Serv Res.

[57] World Health Organization. Health information systems and rehabilitation. Rehabilitation. Geneva: World Health Organization. 2017;2030.

[58] Shirazikhah M, Mirabzadeh A, Sajjadi H, Joghataei MT, Biglarian A, Shahboulaghi FM, et al. Health services coverage: Physical access to rehabilitation facilities in Tehran compare with the country. J Educ Health Promot. 2021;10. 10.4103/jehp.jehp_515_20 PMC7933624 33688513

[59] Stout NL, Binkley JM, Schmitz KH, Andrews K, Hayes SC, Campbell KL (2012). A prospective surveillance model for rehabilitation for women with breast cancer. Cancer.

[60] Garg A, Skempes D, Bickenbach J (2020). Legal and regulatory approaches to rehabilitation planning: A concise overview of current laws and policies addressing access to rehabilitation in five European countries. Int J Environ Res Public Health.

